# Effect of Ciprofloxacin-Loaded Niosomes on *Escherichia coli* and *Staphylococcus aureus* Biofilm Formation

**DOI:** 10.3390/pharmaceutics14122662

**Published:** 2022-11-30

**Authors:** Linda Maurizi, Jacopo Forte, Maria Grazia Ammendolia, Patrizia Nadia Hanieh, Antonietta Lucia Conte, Michela Relucenti, Orlando Donfrancesco, Caterina Ricci, Federica Rinaldi, Carlotta Marianecci, Maria Carafa, Catia Longhi

**Affiliations:** 1 Dipartimento di Sanità Pubblica e Malattie Infettive, Sapienza Università di Roma, Piazzale Aldo Moro, 5-00185 Roma, Italy; 2 Dipartimento di Chimica e Tecnologie del Farmaco, Sapienza Università di Roma, Piazzale Aldo Moro, 5-00185 Roma, Italy; 3 Centro Nazionale Tecnologie Innovative in Sanità Pubblica, Istituto Superiore di Sanità, Viale Regina Elena, 299-00161 Roma, Italy; 4 Dipartimento di Scienze Anatomiche, Istologiche, Medico-Legali e dell’Apparato locomotore, Sapienza Università di Roma, Via Alfonso Borelli, 50-00161 Roma, Italy; 5 Dipartimento di Biotecnologie Mediche e Medicina Traslazionale, Università di Milano, Via Fratelli Cervi, 93-20090 Milano, Italy

**Keywords:** niosomes, drug delivery, ciprofloxacin, anti biofilm activity, bladder cells

## Abstract

Infections caused by bacterial biofilms represent a global health problem, causing considerable patient morbidity and mortality in addition to an economic burden. *Escherichia coli*, *Staphylococcus aureus,* and other medically relevant bacterial strains colonize clinical surfaces and medical devices via biofilm in which bacterial cells are protected from the action of the immune system, disinfectants, and antibiotics. Several approaches have been investigated to inhibit and disperse bacterial biofilms, and the use of drug delivery could represent a fascinating strategy. Ciprofloxacin (CIP), which belongs to the class of fluoroquinolones, has been extensively used against various bacterial infections, and its loading in nanocarriers, such as niosomes, could support the CIP antibiofilm activity. Niosomes, composed of two surfactants (Tween 85 and Span 80) without the presence of cholesterol, are prepared and characterized considering the following features: hydrodynamic diameter, ζ-potential, morphology, vesicle bilayer characteristics, physical-chemical stability, and biological efficacy. The obtained results suggest that: (i) niosomes by surfactants in the absence of cholesterol are formed, can entrap CIP, and are stable over time and in artificial biological media; (ii) the CIP inclusion in nanocarriers increase its stability, with respect to free drug; (iii) niosomes preparations were able to induce a relevant inhibition of biofilm formation.

## 1. Introduction

Biofilms, communities of microorganisms that live in a self-produced extracellular matrix, are an important virulence factor that causes severe problems to public health. Biofilms allow pathogens to escape host defenses and resist antimicrobial treatment [1]. Of particular concern is that biofilm formation on indwelling medical devices can lead to serious, recalcitrant infections [2].

Uropathogenic *Escherichia coli* (UPEC) and *Staphylococcus aureus* are frequently detected in patients with indwelling urinary tract devices [3,4,5,6]. Urinary tract infections (UTIs) are considered to be the most common bacterial infections, affecting around 150–250 million people each year worldwide. These infections account for 75% of infections in community settings and 50–65% of those in healthcare settings [3]. Almost all healthcare-associated UTIs are caused by instrumentation of the urinary tract, whose permanence can lead to complications such as prostatitis, epididymitis, and orchitis in males, and cystitis, pyelonephritis, endocarditis, and meningitis in patients [7,8]. Ciprofloxacin (CIP) is one of the most commonly prescribed fluoroquinolone antibiotics for UTIs to which both *E. coli* and *S. aureus* have become resistant [9,10,11].

Progresses in the field of nanotechnologies applied to therapy and diagnosis have led to the birth of a new branch of science defined as “nanomedicine”, within which a prominent space is occupied by a particular class of unconventional pharmaceutical forms known as pharmaceutical nanocarrier [12].

Nanotechnology has led to the discovery of various types of nanocarriers, which bring several features: favorable physical-chemical features, protection of the loaded active compound, and especially controlled and targeted drug delivery towards the active site decreasing, therefore, unwanted effects of the drug on surrounding tissues, and improving drug half-life in the body [13]. Moreover, the use of nanocarriers as a drug-delivery system thus allows the drug to reach the active site in higher concentration, reducing the needed dosage amount and increasing patient compliance. So, the design of an efficient drug delivery system is fundamental and crucial.

Many delivery modalities find clinical practicality in the field of urology, specifically in the treatment of UTIs, and offer advantages over conventional methods.

Intravesical therapy is a local drug administration, and it is now considered highly promising, providing a high concentration of drugs with the great advantage of minimal systemic side effects. This administration route could be useful to improve the treatment of pathological bladder conditions such as interstitial cystitis, bladder pain syndrome, or urinary infections in which both *E. coli* and *S. aureus* could be involved. Unfortunately, many drugs are not stable in the hostile urine environment, so also, in this case, drug delivery systems, able to protect the loaded drug from degradation, could represent an efficient strategy [14].

Among the various nanocarriers, niosomes are successfully used in different pharmaceutical applications. They are stable, non-immunogenic vesicles suitable for hydrophilic and lipophilic drug loading and delivery. Moreover, niosomes are vesicles produced by the self-assembly of surfactants, which are more stable and less expensive compared to the phospholipids used for liposomes [15].

The most used surfactants for niosomal preparation are the non-ionic ones (which do not possess a charged group in their hydrophilic heads) because they are biocompatible, more stable, and less toxic with respect to the other types of surfactants such as amphoteric, anionic, or cationic ones [16]. Non-ionic surfactants possess a hydrophilic head and a hydrophobic tail, which, when in contact with an aqueous environment, arrange to form a vesicle with a hydrophilic inner core surrounded by one or more concentric lipophilic bilayers.

Recently, the advances in pharmaceutical technology and, in particular, in nanocarrier preparation and characterization provided a promising tool for enhancing the activity and safety of available antimicrobial agents.

Various studies described the antimicrobial and antibiofilm activity of peculiar CIP-loaded niosome formulations against Gram-negative and Gram-positive bacteria [17,18,19] but according to our literature review, only few authors investigated Span 80-Tween 85 based niosomes (from Scopus.com accessed on 20 October 2022).

This study aims to prepare and characterize specific surfactant based nanocarriers entrapping CIP and testing their potential antimicrobial effect against strong biofilm producer strains. Niosomal vesicles composed of Tween 85 and Span 80 (in an equimolar mixture) have been prepared. Both selected surfactants were chosen for the presence, in their chemical structure, of oleic acid (3 molecules in Tween 85 and 1 in Span 80) moiety able to reduce expression of inflammatory molecules. Moreover, the lipophilic bilayer structure of niosomes (administered by intravesical route) could potentially facilitate their adherence to the apical membrane of the vesical surface and enhance CIP efficacy [20]. In addition, Hayashi et al. demonstrate that Span 80 niosomes are able to perturb phospholipid membranes. This could be useful in the interaction of niosomes with the microbial membranes and biofilm environment and in achieving a more efficient internalization of the loaded drug [21,22].

Here, the proposed nanocarriers have been deeply characterized considering several physical-chemical features such as hydrodynamic diameter, ζ-potential, morphological and bilayer characteristics, and biological effectiveness. The antibiofilm activity of CIP-loaded niosomes towards strong biofilm producer bacterial strains was also studied.

## 2. Materials and Methods

### 2.1. Materials

Tween 85, Span 80, ciprofloxacin (CIP), diphenylhexatriene (DPH), pyrene, Hepes, ethanol F.U., methanol, chloroform, were purchased by Sigma-Aldrich (St. Louis, MO, USA).

### 2.2. Preparation of Niosomes and Drug-Loaded Niosomes

The niosomal vesicles were obtained through the film layer preparation technique, better known as Thin Layer Evaporation (TLF) [23]. The components of the vesicles are weighed on the balance according to data reported in Table 1. An organic mixture of chloroform-ethanol 3:1 (*v*/*v*) was useful to solubilize the lipophilic compounds. Subsequently, it is removed under reduced pressure Rotavapor^®^ R-210 (Büchi-Italia S.r.l., Assago (MI), Italy) at room temperature for one hour, leading to a thin layer of film in the test tube. Finally, an oil pump is applied for another hour to remove any residue of the organic solvent. 

Then the sample is hydrated; in the case of “empty’’ niosomes, the hydration is carried out by 5 mL of Hepes buffer (pH = 7.4, M = 0.01), while in the case of “loaded’’ niosomes, 5 mL of a CIP solution (2 mg/mL) are used. A small amount of HCl is used to prepare the CIP solution, to facilitate the solubilization of the drug in Hepes buffer; HCl will then be removed by dialysis (dialysis tube cut-off 10.000 Da) for a period of 3 h.

Subsequently, the vortex action on the sample allows the detachment of the film layer from the wall of the test tube, and a suspension of multilamellar vesicles is obtained. This suspension is then sonicated with an ultrasonic disruptor sonicator (Vibracell-VCX 500, Sonics, Taunton, MA, USA) to obtain unilamellar vesicles (5 min, 65 °C, and 25% amplitude).

In order to remove any impurities or substances not taking part of the vesicular structure, the sample was purified by size exclusion chromatography on glass column of Sephadex G75. Finally, filtration is performed (MF-Millipore^®^, Ireland, E.U. 0.22 μm) in order to retain impurities and to sterilize the sample in accordance with Ph. Eur.

### 2.3. Small Angle X-ray Scattering

Small Angle X-ray Scattering (SAXS) experiments were carried out at the ID02 SAXS beamline of ESRF (Grenoble, France) DOI:10.15151/ESRF-ES-624938971. Purified suspensions were put in Kapton capillaries at room temperature, irradiated with a monochromatic beam, *λ* = 0.1 nm, for short exposure times (0.5–1 s) to avoid radiation damage. The intensity spectra were acquired at two different sample-to-detector distances, namely 1 m and 10 m, and joined after careful background subtraction to obtain the intensity spectra in 0.006 < q < 5 nm^−1^ momentum transfer range, where q = 4*π*sen(ϑ/2)/*λ*, being ϑ the scattering angle. The intensity decay gave information on the internal structure of nano-sized particles [24]. To this end, the profiles were fitted with a core-multishell model that describes the scattering from vesicles and niosomes, with an aqueous spherical core and a layered shell composed of a hydrophobic stratum inserted between two hydrophilic layers [25].

### 2.4. Dynamic Light Scattering (DLS) and ζ-Potential Measurements

The prepared samples were characterized by evaluating: hydrodynamic diameter, ζ-potential and PDI (polydispersity index that gives information on size distribution), employing a Malvern Nano ZS90 apparatus (Malvern Instruments, Worcestershire, UK), equipped with a 5 mW HeNe laser, λ = 632.8 nm. 

The scattering angle was 90°, and the analysis of the intensity autocorrelation function was carried out using the Contin algorithm and analyzed by using the cumulant method to get the values of the particle dimensions and size distribution (PDI) [26]. 

The calculated mean hydrodynamic diameter corresponds to the intensity-weighted average [27]. Electrophoretic mobility of the vesicles was measured by laser Doppler anemometry using the Malvern Zetasizer Nano ZS90 apparatus (Malvern Instruments, Worcestershire, UK). The ζ-potential was obtained by converting the mobility (u) using the Smoluchowski relation ζ = uη/ð, where η is the viscosity and the permittivity of the solvent phase [28].

### 2.5. Transmission Electron Microscopy (TEM)

Morphology of niosomes was obtained by visualizing the samples by TEM analyses. One drop of empty and loaded nanocarriers was placed into a formvar carbon-coated grid. After 2 min adsorption, niosomes were negatively stained with 2% (*v*/*v*) filtered aqueous sodium phosphotungstate acid (PTA) and examined by a FEI 208S transmission electron microscope (FEI Company, Hillsboro, OR, USA) with an accelerating voltage of 100 kV. To optimize image editing, Adobe Photoshop software was used.

### 2.6. Fluorometric Measurements

Information about the lipophilic bilayer of niosomes and CIP-loaded niosomes (samples A and B) was obtained by measuring the DPH fluorescence anisotropy, which is a parameter correlated to membrane rigidity or fluidity [29]. The samples were prepared to dissolve in the organic mixture of the probe (2 × 10^−4^ M), together with the other components, following the same preparation method described in paragraph 2.2. DPH fluorescent measurements were performed using a luminescence spectrometer (LS5013, PerkinElmer, Waltham, MA, USA) with excitation λ ex = 350 nm and detecting the fluorescence intensity at λ em = 428 nm [30]. In employing Equation (1), the fluorescence anisotropy (r) was determined:

(1) $$\mathrm{Fluorescence}\;\mathrm{anisotropy}\;\left(r\right)=\frac{\left(I_{\mathrm{vv}\;}-I_{\mathrm{vh}\;}\right)\times \;G}{(I_{\mathrm{vv}}+2I_{\mathrm{vh}})\times \;G}$$

where I_vv_, I_vh_, I_hv_, and I_hh_ are fluorescent intensities, subscript v (vertical) and h (horizontal) represent the orientation of polarized light, and *G* = I_hv_/I_hh_ factor is the ratio of sensitivity of the detection system for vertically and horizontally polarized light. 

Additionally, bilayer characterization studies were also performed utilizing a different fluorescent probe: the pyrene. This probe is useful to evaluate the polarity and microviscosity of the vesicular bilayer and was used both in empty niosomes and in CIP-loaded ones. Samples A and B were prepared by adding Pyrene (4 mM) to the components (following the same preparation method described above). By fluorescence measurements, it is possible to investigate the lateral distribution and the mobility of the membrane compounds. Pyrene is a fluorescence probe with a spectrum characterized by five emission peaks as the monomer (from I1 to I5) and one as excimer (IE). In particular, the ratio I1/I3, corresponding to the first and third vibration bands of the Pyrene spectrum, is related to the polarity of the probe environment. Pyrene can form intramolecular excimer based on the viscosity of the probe microenvironment [31].

### 2.7. Drugs Entrapment Efficiency (EE%)

Utilizing the UV-vis spectrophotometer (Lambda 25, PerkinElmer, Waltham, MA, USA), the entrapment efficiency (E.E.) of CIP inside niosomes was determined. In particular, the CIP entrapped amount was calculated by using the calibration curve previously defined. Loaded niosomes were diluted in Hepes buffer, and the absorbance of drugs at λ = 271 nm was measured [32].

E. E. % was calculated as (2):

(2) $$E.E.\;\left(\%\right)=\frac{\mathrm{Entrapped}\;\mathrm{drug}\;\left(\mathrm{mg}\right)}{\mathrm{Total}\;\mathrm{drug}\;\mathrm{used}\;\left(\mathrm{mg}\right)}\times 100$$

### 2.8. Physicochemical Stability

Both unloaded and loaded niosomes were stored at two different temperatures: room temperature/4 °C for a period of 90 days. The data concerning nanocarrier stability were collected employing DLS (Malvern Instruments, Worcestershire, UK).

These experiments consist of monitoring over time the dimension and ζ-potential variations of the samples by DLS measurements.

Empty and loaded niosomes were also subject to stability studies performed in simulated biological fluids (Artificial Urine, pH 6.6) in order to evaluate the niosomal stability. Artificial urine was prepared according to Monika Pietrzyńska et al., 2017 [33], and the composition is reported in Table 2.

These tests were executed by carrying out DLS measurements to assess that the size, the PDI, and the ζ-potential of the vesicular suspensions remained constant.

In order to carry out these experiments, 1 mL of the sample was added to 1 mL of the artificial body urine and put into a test tube, subject to a magnetic stirrer at 37 °C to mimic the body temperature. This experiment lasted 24 h.

Moreover, free CIP and CIP-loaded niosomes were evaluated over time, for a period of 90 days, at two different storage temperatures (25 °C and 4 °C).

This experiment consists of monitoring over time the drug stability by means of a UV-vis spectrophotometer, observing the intensity and shape of the peak at 271 nm [32].

### 2.9. In Vitro Release Studies

In vitro drug release experiments were carried out by inserting in a dialysis tube (molecular weight cut-off: 8000 MW by Spectra/Por^®^) the drug-loaded niosomal suspension. The dialysis tube was immersed in the release medium (Hepes Buffer 10 mM, pH 7.4 or Artificial Urine, pH 6.6) at 37 °C and gently magnetically stirred during the experiment.

The drug concentration amount in the release medium was detected by UV spectrophotometer (Lambda 25, PerkinElmer, Waltham, MA, USA) at different time points until up 48 h. In order to perform UV analysis, 1ml di external medium was withdrawn and immediately analyzed to the spectrophotometer and then re-inserted back.

Reported values represent the mean values over three repeated independent experiments, and errors are the standard deviation.

### 2.10. Bacterial Strains

Uropathogenic *Escherichia coli* ATCC 700928 (CFT073) and *Staphylococcus aureus* ATCC 6538P were biofilm-forming reference strains obtained from the American Type Culture Collection (ATCC, Manassas, VA, USA). *E. coli* K-12 MG1655 was a weak biofilm producer strain. The microorganisms were grown in Brain Heart Infusion broth (BHI) (Oxoid) and stored in 15% glycerol-BHI at −80 °C.

### 2.11. Determination of Minimum Inhibitory Concentration (MIC) of CIP-Loaded Niosomes

The MIC determination of CIP-loaded niosomes was performed by the microdilution method and carried out in triplicate. Exponentially growing bacterial cultures were diluted to cell density 0.5 McFarland, and 10 μL of bacterial suspension was added to 190 µL of BHI (Oxoid) containing CIP-loaded in niosomes at concentrations from 125 µg/mL to 7.8 µg/mL. Empty niosomes were diluted and tested similarly. After the incubation at 37 °C for 24 h, the bacterial growth was evaluated by measuring the optical density at 595 nm. All experiments were conducted in triplicate.

### 2.12. Effect of CIP-Loaded Niosomes on Bacterial Biofilm Production

A volume of 20 µL of each bacterial strain (1–2 × 10^8^ CFU/mL) was inoculated into wells of a 96- well polystyrene plate containing 180 μL of Tryptic Soy Broth (TSB) and incubated for a period of 24 h at 37 °C. Then, after washing with phosphate-buffered saline, the plates were allowed to dry. The wells were stained for 15 min with crystal violet (Sigma-Aldrich, 1% *w*/*v*), a basic dye that binds negatively charged molecules. The dye was solubilized with 95% (*v*/*v*) ethanol for 30 min. The optical density (OD) at 570 nm of each well was measured, and biofilm production was classified as described by Stepanovic et al., 2004 [34]. Based on the cut-off OD, defined as three standard deviations above the mean OD of the negative control (ODc), strains were classified as follows: OD ≤ ODc = no biofilm producers, ODc < OD ≤ (2 × ODc) = weak biofilm producers, (2 × ODc) < OD ≤ (4 × ODc) = moderate biofilm producers, and (4 × ODc) < OD = strong biofilm producers. 

In order to measure the biofilm inhibition induced by CIP-loaded niosomes, the growth medium was supplemented with these substances at a concentration of 3.9 µg/mL. The inhibition of cell attachment was evaluated after 24 h incubation at 37 °C. The percentage of biofilm inhibition by sub-MIC niosomes preparation has been calculated using the following formula [35]:

𝐵𝑖𝑜𝑓𝑖𝑙𝑚 𝑖𝑛*h*𝑖𝑏𝑖𝑡𝑖𝑜𝑛 (%) = 100 − (𝑂𝐷570 𝑠𝑎𝑚𝑝𝑙𝑒/𝑂𝐷570 𝑐𝑜𝑛𝑡𝑟𝑜𝑙 × 100)

Values higher than 40% were considered relevant in biofilm inhibition. Uninoculated TSB broth was used as a negative control. Experiments were run in sextuplicate.

### 2.13. SEM Analysis

Samples of biofilms grown on aluminum stubs were washed and fixed in 2.5% glutaraldehyde in 0.1 M phosphate buffer pH 7.4 for at least 48 h. Samples were washed overnight in phosphate buffer pH 7.4, and the day after, they were postfixed with OsO_4_ 1.33 % in H_2_O for 1 h at room temperature. Samples were then washed for 20 min with H_2_O, and then they were treated for 30 min with tannic acid 1% in H_2_O. Samples were then washed for 20 min with H_2_O. The excess water was dried carefully with filter paper, and then the samples were mounted on the specimen holder and observed in a Hitachi SU3500 microscope (Hitachi, Japan) at variable pressure conditions of 5 kV and 30 Pa [36,37]. Three-dimensional reconstruction of images was undertaken by Hitachi Map 3D Software (v.8.2., Digital surf, Besançon, France). For each image, a selected area was extracted for the 3D image reconstruction procedure. The surface topography of the extracted area is shown in false colors [38], and it was processed by the particle count procedure to evaluate the size of ECM granules.

### 2.14. Evaluation of Intracellular Uptake

In order to visualize niosomes intracellular uptake, T24 cells were seeded on 8-well chamber-slides (Falcon) for 24 h at 37 °C and exposed to niosomes loaded with Nile Red dye/CIP-Nile Red co-loaded niosomes [39], for different times. Cells treated with free Nile Red, prepared as 1 mg/mL stock solution in acetone and used at a final concentration of 100 ng/mL, were used as control. After the incubation times, cells were washed with phosphate-buffered saline solution (PBS) at pH 7.4 and fixed in methanol/acetone (1:1) for 5 min at −20 °C. Slides, extensively washed with PBS, were mounted with 0.1% (*w*/*v*) p-phenylenediamine in 10% (*v*/*v*) PBS, 90% (*v*/*v*) glycerol, pH 8.0 and observed by fluorescence microscopy using a Leica DM4000 (Leica Microsystem, Wetzlar, Germany) fluorescence microscope, equipped with an FX 340 digital camera.

### 2.15. Cytotoxicity Studies

T24 cells (concentration of 1 × 10^4^/well) were seeded in 96-well plates and cultured at 37 °C with 5% CO_2_ for 24 h. Different concentrations of CIP-niosomes (from 250 µg/mL to 31.2 µg/mL) were added to cell monolayers and incubated for 24 h. Then, 100 μL of 0.5 mg/mL of 3-(4,5-dimethylthiazol-2-yl)-2,5-diphenyltetrazolium bromide (MTT) reagent was added to each well for an additional 4 h. Afterwards the dye was eluted with 200 μL of DMSO for 10 min at room temperature and, finally the OD at 568 nm was measured using a microplate reader (PerkinElmer, Boston, MA, USA).

### 2.16. Statistical Analysis

For the statistical analysis of niosomes characterization studies, two-way ANOVA was performed. Multiple comparisons were performed according to Tukey’s test for ζ-potential, hydrodynamic diameter, and polydispersity index (PDI), respectively, and Dunnett’s test for the Turbidimetric assay. Any *p*-value < 0.05 was considered statistically significant. For the cytotoxicity assay and antimicrobial studies, all values were reported as mean ± standard deviation (SD). Statistical analyses were performed by one-way ANOVA followed by Tukey’s post hoc pairwise tests (Graph Pad Prism, Version 5.0). A *p*-value of less than 0.05 (* *p* < 0.05) was considered significant.

## 3. Results and Discussion

### 3.1. Characterization of Empty and Loaded Niosomes

**Figure 1:**
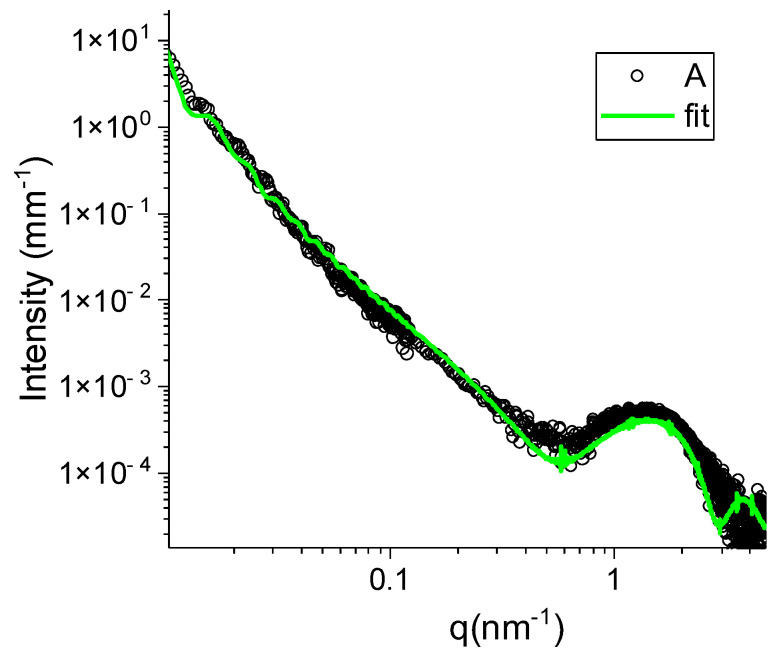
SAXS spectrum of empty niosomes, sample A (22.5 mM, room temperature, open dots) reported in log-log scale. The green line is the best fit.

Empty and loaded niosomes were deeply characterized. First of all, preliminary SAXS analyses have been useful in evaluating the ability of surfactants to form niosomes, even if in the absence of cholesterol. SAXS techniques represent a non-invasive tool to provide ensemble-averaged and, thus, statistically relevant information on the structure and conformation of materials. Even though the samples are in a very dilute solution, the high brilliance of the synchrotron radiation allows for obtaining good statistics of the data in a wide range of q, with the uncertainty on the measure intrinsically represented by the fluctuation of the scattered data. Figure 1 reports the intensity spectrum measured for the suspension composed by Tween 85: Span 80 at a 1:1 molar ratio after purification. The intensity profile is characteristic for quite monodisperse nano-sized particles that have been modeled with a spherical aqueous core (size ≥ 100 nm) surrounded by a single surfactant bilayer (with a hydrophobic core between two hydrophilic regions) of about 5 nm thickness, demonstrating the ability of the selected surfactants to form unilamellar niosomes.

Moreover, empty and loaded niosomes were characterized by evaluating hydrodynamic diameter, ζ-potential, PDI, and CIP entrapment efficiency (EE%,) and the results have been reported in Table 3. In particular, dynamic light scattering analysis showed that CIP-niosomes are characterized by an increased size with respect to empty ones (from 113.10 nm to 260 nm) that confirm the drug inclusion inside the vesicles [40], while the ζ-potential remains constant and enough negative to assure colloidal stability. As previously described, the polydispersity index (PDI) is an important parameter to be taken into account. Both formulations, loaded and unloaded, showed a PDI of 0.2, which indicates monodisperse samples. Probably, niosomes characterized by a low PDI value could be stable over time, and, in terms of drug pharmacokinetics, they could be characterized by similar behavior after in vivo administration [41].

**Figure 2:**
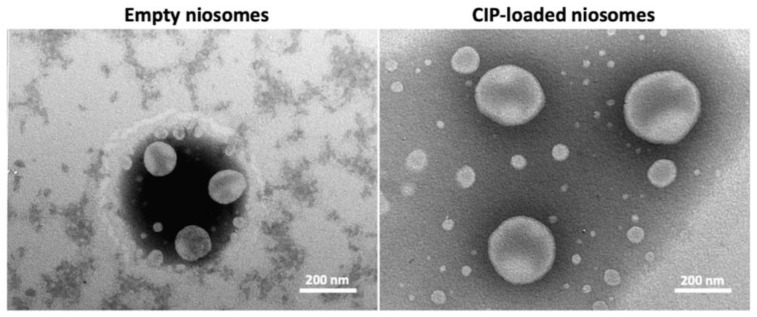
Transmission electron microscopy images of empty and CIP-loaded niosomes.

Electronic visualization of empty and CIP-loaded niosomes showed spherical vesicles with size corresponding approximately to dimensions revealed by DLS. The increased size of CIP-loaded niosomes reported by DLS was confirmed by electronic observations that also revealed no changes in a spherical shape (Figure 2).

From Table 3, it is possible to observe that the CIP EE%, obtained by UV analysis, is almost 20% (0.4 mg/mL), which is a concentration useful for biofilm treatment, as demonstrated by the results.

In order to characterize the lipophilic bilayer of empty and loaded niosomes, the microviscosity, polarity, and anisotropy were studied. Following the method previously described, the nanocarriers have been prepared to load the molecular probe (pyrene) inside the vesicles. The values of polarity and microviscosity have been collected to analyze the obtained fluorescence spectrum, and the values of polarity and microviscosity have been collected (Table 4). In particular, the polarity values were quite similar for niosomes (A) and CIP-niosomes (B), but microviscosity values decreased when CIP was loaded in the nanocarrier. The decreased microviscosity could be explained by a partial CIP localization in the niosomal lipophilic compartment. In fact, in agreement with the drug’s chemical structure, the CIP lipophilic portion could be located inside the bilayer, while the hydrophilic portion could be inside the aqueous core. According to these results, the anisotropy values increase with drug inclusion. The anisotropy value suggests a more rigid bilayer of the loaded niosomes with respect to empty ones to confirm the partial drug localization in the bilayer [42].

### 3.2. Stability Studies

#### 3.2.1. Physical Stability of Niosomes

**Figure 3:**
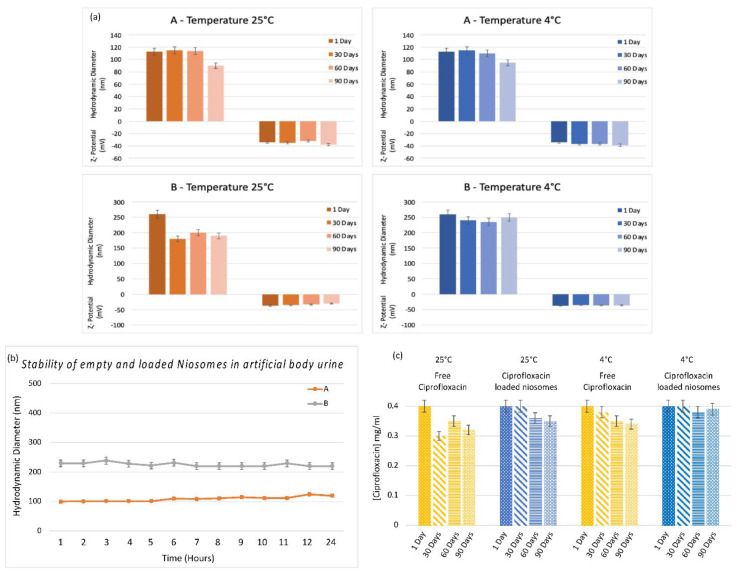
(**a**) Result of investigation on physicochemical stability of empty niosomes (A) and CIP-loaded niosomes (B) in terms of hydrodynamic diameter and ζ-potential until up 90 days at 4 °C and room temperature. (**b**) Stability studies in the presence of artificial body urine, following variation of hydrodynamic diameter and ζ-potential values of CIP-loaded niosomes; Pre-exp values refer to CIP-loaded niosomes before the presence of artificial body urine. (**c**) Stability studies over time of free CIP and CIP-loaded into niosomes at two different storage temperatures over a 90-day period.

Studies on physical stability were also performed according to the method previously described, and the obtained data are reported in Figure 3. In particular, both samples (empty and loaded niosomes) appeared to be stable for 90 days when stored at 4 °C (no significant dimensional change is observed but only minor variations), while the hydrodynamic diameter of sample A decreased when it was stored at room temperature (Figure 3, Panel a). So, it is possible to conclude that the samples have been characterized by significant colloidal stability when maintained at 4 °C [43].

#### 3.2.2. Stability of Niosomes in Artificial Urine

The stability of samples A and B was also studied in artificial urine (Figure 3, Panel b) to mimic the effect of the media on the vesicle stability after intravesical administration. The experiments were carried out at 37 °C evaluating the vesicle size by DLS measurements for 24 h as described in the Material and Methods section (Section 2). During the experiment, no significant changes in hydrodynamic diameter were observed for both samples (empty and loaded vesicles). It is possible to conclude that the artificial media doesn’t affect the niosome integrity. 

#### 3.2.3. Stability Studies over Time of Free CIP and CIP-Loaded into Niosomes

In order to evaluate CIP stability in terms of decomposition or degradation, the unloaded and loaded drug peak was observed by a UV spectrophotometer. The UV spectra were recorded immediately after sample preparation and after 30, 60, and 90 days at room temperature and 4 °C. The CIP concentration values obtained by these experiments were reported in Figure 3, Panel c. Drug stability (at room temperature and 4 °C) has been enhanced by its inclusion in the vesicles with respect to free drug dissolved in Hepes solution. 

### 3.3. CIP Release Studies

**Figure 4:**
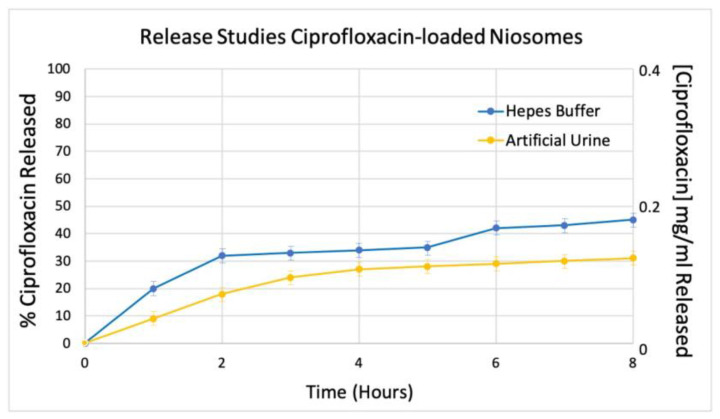
CIP release profile until up 24 h. Data were obtained as the mean of three independent experiments.

*In vitro* release studies of niosomal loaded sample in Hepes buffer and artificial urine have been carried out, and data obtained are shown in Figure 4. The total amount of released drug by niosomes is around 50% in Hepes Buffer and around 40% in simulated biological fluid. From these data, it is possible to observe that Sample B is characterized by the same release profiles in both media. Probably, these results are related to physical-chemical features of sample B. In fact, the anisotropy value (A = 0.35) of loaded niosomes could suggest that the CIP is retained by the bilayer vesicles due to their rigidity. Moreover, according to Uhljar et al., 2021 and Volpe 2004, CIP is characterized by a slow drug permeation coefficient (Pbl) that could prevent complete drug diffusion through the vesicle bilayer [44,45]. Concerning the integrity of niosomes, it is possible to affirm (by DLS measurements) that the niosomal formulations are characterized by no vesicle degradation at the experimental condition.

### 3.4. In Vitro Antibacterial Activity of CIP-Loaded Niosomes

**Figure 5:**
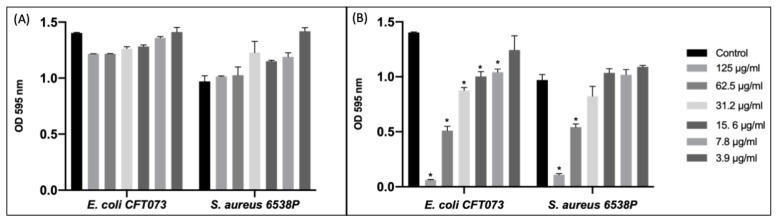
Susceptibility test with empty niosomes (**A**) and CIP-loaded niosomes (**B**). Data were expressed as mean ± SD. All considered conditions were compared to untreated control. * p value ≤ 0.05.

In our study, the blank niosomal preparations showed no or lower antimicrobial activity compared to CIP-loaded niosomes (Figure 5A).

CIP-loaded niosomes significantly inhibited the growth of *E. coli* more than the *S. aureus* strain (Figure 5B). Probably, niosomal vesicles, thanks to specific surfactants employed, are able to perturb microbial phospholipid membranes interacting efficiently with Gram-negative bacteria. Furthermore, since fluoroquinolones can cross the cytoplasmic membrane by simple diffusion, any condition which creates a concentration gradient towards the bacterial cell outer membrane could improve drug permeation [46,47,48].

### 3.5. Biofilm Production and Anti-Biofilm Activity of the Sub-MIC of Formulated Niosomes

The biofilm production ability of bacterial strains was classified into four groups: no biofilm, weak, moderate, and strong. In our experimental conditions, *E. coli* CFT073 and *S. aureus* 6538P were confirmed as strong biofilm producers [49,50].

In order to verify the effect of CIP-loaded niosomes on biofilm production, we treated bacterial strains with encapsulated CIP for 24 h. The results obtained showed that the sub-MIC concentration of niosome-encapsulated CIP significantly inhibited biofilm formation when compared with empty niosomes (Table 5).

The antibiofilm activity of antibiotic-loaded niosome preparations against a wide variety of pathogens was previously reported [51,52,53,54].

A significant decrease in *S. aureus* biofilm formation by niosome-encapsulated CIP was described by some authors [19]. They suggested the adsorption of the nanocarriers on the biofilm surface with subsequent delivery of the encapsulated drug to the bacterial cells. Moreover, their study, using Real Time PCR, revealed the down-regulation of *ica*B biofilm gene expression compared to the free CIP. They postulated that the reduction of the *ica*B gene expression could be associated with the inhibition of transcription of bacterial genes by reactive oxygen species (ROS) and/or direct interaction of the niosome-encapsulated CIP with the transcription factors involved with the expression of *ica*B.

Dong et al., 2019 revealed that treatment with sub-MICs of CIP for 24 h inhibited biofilm formation and reduced the expression of virulence genes and biofilm formation genes in *E. coli* [55]. Our results, demonstrating the ability to efficiently release the antibiotic during the biofilm formation process, confirmed that CIP-loaded niosome formulation represents a suitable drug delivery system.

### 3.6. Ultrastructural Morphology

As previously described by other authors for the CIP [55], SEM analyses confirmed that the biofilm structure of the *E. coli* changed significantly after treatment with sub-MICs of CIP-loaded niosomes.

**Figure 6:**
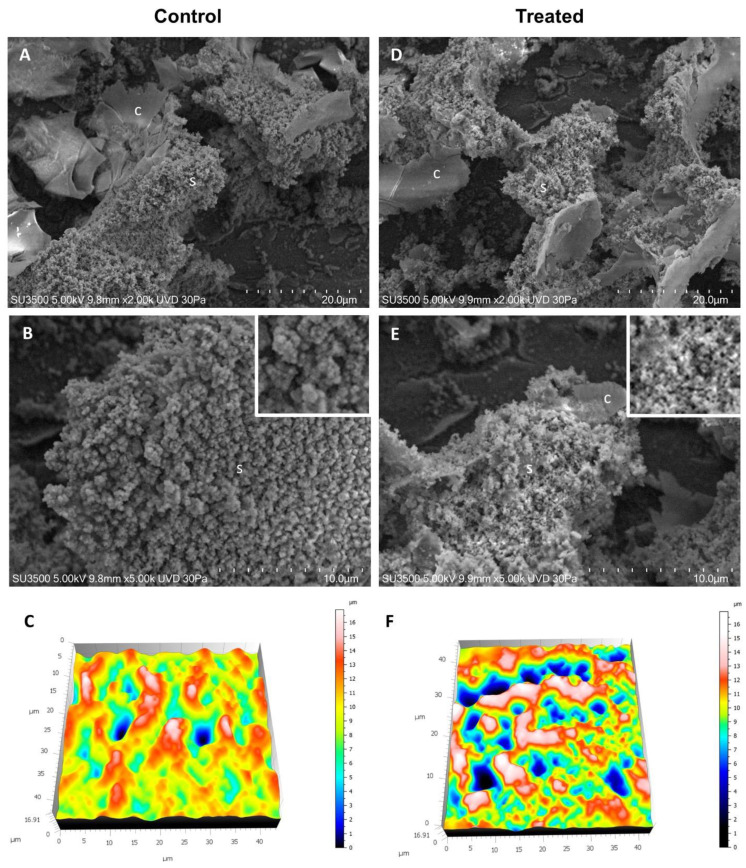
VP-SEM images of *E. coli* (**A**–**C**) and *E. coli* treated with CIP-loaded niosomes (**D**–**F**). (**A**) Low magnification (2.00K), ECM shows compact and smooth ECM areas (c), as well as spongy and rough areas (c). (**B**) Higher magnification (5.00K) of the ECM spongy area, ECM trabeculae show a globular structure (inset). (**C**) 3D reconstruction of sample surface topography, ECM trabeculae are represented in white and red areas, and the channels that perforate the ECM are represented with color shades from green to blue. (**D**) Low magnification (2.00K), ECM shows both compact and smooth ECM areas (c), both spongy and rough areas. (**E**) Higher magnification (5.00K) of the ECM spongy area, ECM trabeculae show a fine filamentous network structure (inset). (**F**) 3D reconstruction of sample surface topography, ECM trabeculae are represented in white and red areas, and the channels that perforate the ECM are represented with color shades from green to blue. Note that ECM presents more channels than the control, and trabeculae are thinner than the control sample.

The observation of untreated samples (Figure 6A–C) showed that the sample is characterized by the presence of an abundant extracellular matrix (ECM), whose surface appears irregular, being in some areas smooth and compact (c), while in others it appears spongy and rough (s). At higher magnification (B), it was possible to appreciate the ECM ultrastructure in detail. It consists of a 3D network of trabeculae showing a globular aspect (insert). A labyrinthic system of narrow channels develops in the ECM, giving it a spongy appearance. In Figure 6C, the 3D reconstruction of the sample surface topography is represented. White and red areas are ECM trabeculae, formed by globular structures, and the channels perforating the ECM are represented with color shades from green to blue. When the sample is treated with niosome preparation, as illustrated in Figure 6D–F, ultrastructural modifications appear. The picture at low magnification (D) shows the sample presenting a spongy appearance (s) with also compact and smooth areas (c). However, the image at higher magnification (E) clarifies that the spongy areas have a different meshes structure concerning the untreated sample. The treatment affects the trabecular structure disassembling the globular structures into fine filamentous structures. The change is visible in Figure 6F, where the 3D reconstruction of sample surface topography is presented. A lot of channel openings are visible (blue-green areas), which means that the ECM is more perforated. White and red areas are ECM trabeculae, thinner than those of control samples, due to the disassembling action of the treatment.

**Figure 7:**
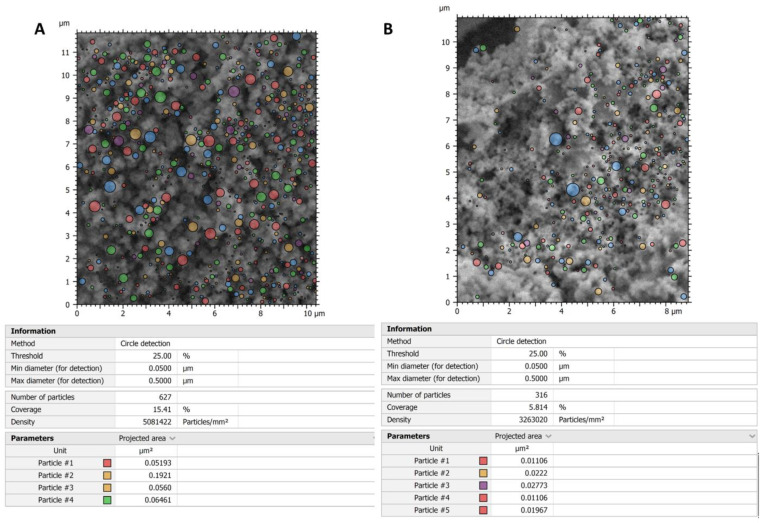
Analysis of globular structures in control (**A**) vs. treated samples (**B**). The particle analysis tool of Hitachi 3D map software revealed that the globular structures in the control sample are about twice those of the treated sample. This may be explained by thinking of a disassembling effect of the treatment on the filament that forms the ECM trabeculae.

To further quantitatively analyze the effect of niosomes on *E. coli* ECM ultrastructure, we used the Hitachi Map 3D Software (v.8.2., Digital surf, Besançon, France) particle analysis tool to detect globular structures that characterize the trabecular system of the control sample and observe if any difference exists in the treated sample. As shown in Figure 7, the control sample has a number of globular structures, sized 0.05–0.5 µm, which is about double that of the treated sample. This can be explained as a disassembling effect of the treatment. In the control, the ECM filaments are tightly coiled, and the treatment breaks down this compact arrangement producing an ECM with a loose filament arrangement.

### 3.7. Evaluation of Intracellular Uptake

**Figure 8:**
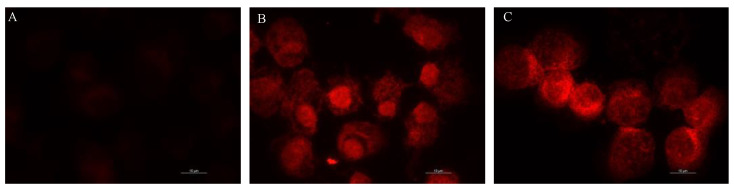
Untreated T-24 cells (**A**), cells treated for 7h with Nile Red loaded niosomes (**B**) and Nile Red and CIP co-loaded niosomes (**C**).

In order to verify the ability to enter epithelial cell monolayers, the CIP-loaded niosomes were probed by following the aggregation of the Nile Red. A fluorescence microscope was used to assess intracellular uptake efficiency. As shown in Figure 8, at 7 h post-treatment, an increased fluorescence in the cells exposed to the CIP-loaded niosomes, with respect to Nile Red niosomes, was detected.

### 3.8. Cytotoxicity Studies

**Figure 9:**
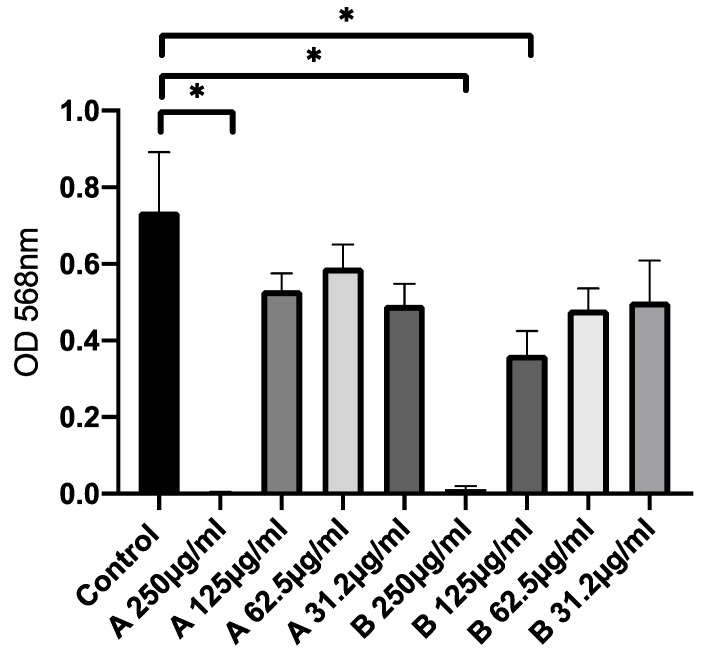
Cytotoxic activity of CIP-loaded niosomes (OD values). MTT assay on human bladder cancer cells (T24). Data were expressed as mean ± SD. All considered conditions were compared to untreated control. * *p* value ≤ 0.05.

In order to evaluate the cytotoxicity activity of these formulations, an MTT assay was performed, incubating niosomal preparations for 24 h with cell monolayers (Figure 9). The treatment with niosomes triggers a cytotoxic effect compared to the untreated control, only at the highest concentration used: 250 µg/mL (for both empty and CIP-niosomes). Moreover, a slight increase in cytotoxicity was observed for CIP-niosomes at the concentration of 125 µg/mL, suggesting the ability of niosomes to target and release the drug. Results obtained confirmed CIP cytotoxicity on T24 cells as well as for other authors [56,57].

## 4. Conclusions

Biofilm-related infections remain a serious concern in clinical services. Considering the impact of infectious diseases on human health, the development of advanced delivery systems able to target drugs directly to the site of interest appears to be a priority in the pharmaceutical area. For this purpose, empty and loaded niosomal formulations (composed of surfactant without cholesterol) were prepared and deeply characterized. Results obtained by physical-chemical characterization and by in vitro functional assays demonstrated that CIP-niosomes might be good candidates for the proposed application.

In fact, niosomal formulations exhibited: (i) good stability over time and in a simulated biological fluid, (ii) the capability to protect the entrapped drug by degradation phenomena, and (iii) controlled CIP release and antibiofilm effects against both Gram-positive and Gram-negative strains. In addition, the niosomes showed reduced toxicity on cell monolayers. The results of this study will contribute to developing niosomal formulations that, employed at the appropriate concentration, could represent a promising drug delivery system, stable and inexpensive (due to the low cost of surfactants) to contrast the biofilm development in bacterial infections.

Further investigations will be necessary to assess the safety and efficacy of the niosomes for a potential application in clinical trials.

## Figures and Tables

**Table 1:** Sample compositions.

Sample	Tween 85 (mM)	Span 80 (mM)	Ciprofloxacin (mg/mL)
A	22.5	22.5	-
B	22.5	22.5	2

**Table 2:** Artificial body urine (pH 6.6): chemical composition.

Reagent	Dosage (g)
Urea	25.0
NaCl	9.0
NH4CL	3.0
Creatinine	2.0
Na2HPO4	2.5
KH2PO4	2.5
Na2SO3	3.0
Distilled water	Total 1.0 L

**Table 3:** Niosomal formulations characterization: summary of physicochemical features.

Sample	Hydrodynamic Diameter (nm) ± SD	ζ-Potential (mV) ± SD	PDI ± SD	Ciprofloxacin (mg/mL)	Ciprofloxacin E.E. %
A	113.10 ± 3.17	−34.80 ± 1.91	0.20 ± 0.01	-	-
B	260.41 ± 4.03	−37.50 ± 2.12	0.20 ± 0.01	0.40	20

**Table 4:** Bilayer characterization. (SD values are all in the range ± 0.01–0.02).

Sample	I_1_/I_3_ (Polarity)	I_E_/I_3_ (Microviscosity)	Anisotropy A.U. (Fluidity)
A	0.96	1.01	0.22
B	0.97	0.46	0.35

**Table 5:** Percentage of biofilm inhibition by sub-MIC niosomes. Values higher than 40% were considered relevant in biofilm inhibition.

Strains	A	B	Free CIP ½ MIC	Free CIP ¼ MIC
*E. coli* CFT073	12.1	64.6	3.8	0.4
*S. aureus* ATCC 6538P	26.3	75.0	9.7	9.7

## Data Availability

Not applicable.
